# Elevated serum IgA following vaccination against SARS-CoV-2 in a cohort of high-risk first responders

**DOI:** 10.1038/s41598-022-19095-7

**Published:** 2022-09-02

**Authors:** Brian T. Montague, Matthew F. Wipperman, Erica Chio, Rowena Crow, Andrea T. Hooper, Meagan P. O’Brien, Eric A. F. Simões

**Affiliations:** 1 University of Colorado School of Medicine, Aurora, CO USA; 2 Regeneron Pharmaceuticals, Inc., Tarrytown, NY USA

**Keywords:** Viral infection, Epidemiology, Health occupations, Viral infection

## Abstract

IgA plays an important early neutralizing role after SARS-CoV-2 infection. Systemically administered vaccines typically produce an IgM/IgG predominant response. We evaluated the serum anti-spike (anti-S) IgG, anti-nucleocapsid (anti-N) IgG and anti-S IgA response following vaccination against SARS-CoV-2 in a cohort of first-responders. Among the 378 completely vaccinated participants, 98% were positive for anti-S IgG and 96% were positive for anti-S IgA. Nine percent were positive for anti-N IgG suggesting prior exposure to SARS-CoV-2. No statistically significant difference was seen in IgA response based on prior evidence infection (p = 0.18). Ninety-eight of those receiving the Moderna vaccine (98%) were positive for anti-S IgA as compared to 91% of those who received the Pfizer vaccine (p = 0.0009). The high proportion of participants observed to have a positive anti-S IgA response after vaccination suggests that the vaccines elicit a systemic response characterized by elevated levels of both IgG and IgA.

## Introduction

IgA has been shown to have an important early role in the neutralization of SARS-CoV-2 virus after infection^1–4^. In serosurveys, IgA positivity has been identified in individuals without detectable IgG and without known history of symptomatic illness^5,6^. Prior studies have suggested that IgA positivity with transient or absent IgG positivity may be seen in individuals with mild or asymptomatic infection^6,7^. In several participants, mucosal IgA secretions were demonstrable in individuals without detectable IgA or IgG in the serum^6^.

There are now multiple FDA emergency use authorized vaccines with demonstrated high level of efficacy in preventing severe COVID-19 disease^8^. Among the most potent COVID-19 vaccines are the mRNA type vaccines manufactured by Moderna and Pfizer BioNTech with studies showing efficacy in prevention of symptomatic illness as high as 90–95%. While most of the available vaccines protect well against severe illness, more variability has been seen in the ability of the vaccines to prevent symptomatic infection with rates ranging from 50–90%.

Traditionally, intramuscularly or intradermally administered vaccines are thought to generate strong IgM and IgG predominant responses which provide strong protection against lower respiratory tract disease^4^. Though the response may be less strong, there is data that in some cases systemic administration of vaccines can elicit the production and release of secretory IgA^4^. Serial immunization against influenza has been shown to elicit both IgG and IgA responses, potentially reflecting boosting of previously developed immunity^9^. In evaluation of the immune response to influenza, IgA together with IgG has been found to be more important in protection against secondary infection whereas IgG and IgM predominate in the primary immune response^10^. There is limited data showing both increased positivity for anti-SARS-CoV-2 IgA in the serum and mucosal secretions following immunization^11^.

To evaluate the impact of prior SARS CoV-2 exposure and illness on IgA response after vaccination, we evaluated the change in serum anti-SARS-CoV-2 IgA following receipt of COVID-19 vaccination as part of a longitudinal serosurvey of first responders at high risk for SARS-Cov-2 infection.

## Results

A total 1007 participants underwent baseline screening and sample collection, 779 had follow-up testing at 2–3 months and 619 follow-up testing at 6 months. Four hundred fourteen (41%) worked as fire fighters, 241 (24%) as emergency medical service providers, 201 (20%) as police and 151 (15%) in other positions related to one of the participating agencies. The median age of participants was 42 with interquartile range of 17. Seventy-six percent were male. Nine hundred six (92%) identified as white and 870 (90%) non-Hispanic. The cohort was overall healthy with 16 (2%) reporting a history of diabetes, 11 (1%) heart disease, 2 (< 1%) chronic kidney disease, and 74 (7%) asthma. Thirteen participants (1%) reported taking an immune suppressant and 1 participant (< 1%) reported receipt of a solid organ transplant. A total of 486 participants had at least one documented COVID-19 vaccination 14 days or more prior to a lab collection (see Table 1). Among these, 161 (33%) specified receipt of the Pfizer BioNTech vaccine, 321 (66%) Moderna, 1 (< 1%) Janssen, and 3 (< 1%) unspecified. Three hundred seventy-eight (78%) had completed a full vaccine series and 108 (22%) had completed a partial series.

**Table 1:** Serologic positivity at follow-up by vaccine received and category of SARS-CoV-2 exposure and infection.

	Anti-S IgA	Anti-S IgG	Anti-N IgG
COVID-19 vaccine specified*			
Pfizer (n = 161)	149 (92%)	155 (96%)	15 (9%)
Moderna (n = 321)	301 (94%)	304 (95%)	30 (9%)
**Vaccine status**			
Complete (n = 378)	364 (96%)	373 (99%)	35 (9%)
Partial (n = 108)	89 (82%)	90 (83%)	10 (9%)
Naïve (n = 520)	72 (14%)	66 (12%)	30 (6%)
SARS-CoV-2 exposure/infection**			
Definite COVID-19 (n = 58)	56 (97%)	55 (95%)	20 (34%)
Probable COVID-19 (n = 69)	65 (94%)	69 (100%)	8 (12%)
Possible COVID-19 (n = 24)	23 (96%)	22 (92%)	17 (71%)
Definite asymptomatic infection (n = 6)	6 (100%)	6 (100%)	–
Possible asymptomatic infection (n = 31)	29 (94%)	29 (94%)	–
No evidence of SARS-CoV-2 infection (n = 298)	274 (92%)	282 (95%)	–
Prior SARS-CoV-2 infection (n = 136)*^,^**	127 (93%)	130 (96%)	28 (21%)

*One participant received the Janssen vaccine and 3 did not specify. Given the small number those were not included.

**Prior SARS-CoV-2 infection was defined as either definite COVID-19, probable COVID-19 or definite asymptomatic infection.

Three-hundred seventy-three (99%) of the 378 completely vaccinated were positive for anti-S IgG as compared to 90 (83%) of those partially vaccinated (p < 0.0001). No statistically significant difference was observed in anti-N IgG response with 35 (9%) positive among those completely vaccinated as compared to 10 (9%) positive among those partially vaccinated. Twenty-eight of the 133 (21%) with evidence of prior infection were positive for anti-N IgG as compared to 17 of the 353 (5%) with no evidence of prior infection (p < 0.0001). There was no statistically significant difference in anti-S IgG response with 130 out of 133 (98%) with evidence of prior infection positive as compared 274 (92%) of the 298 without evidence of prior infection (p = 0.25). The median anti-S IgG ratio was higher at 8.7 for those with a history of infection as compared to 8.2 for those without (p < 0.0001).

Three hundred sixty-four (96%) of the 378 completely vaccinated were positive for IgA as compared to 89 (82%) of the 108 partially vaccinated (p < 0.0001). The median anti-S IgA ratio was higher at 7.6 for those with a history of infection as compared to 4.8 for those without (p < 0.0001). Complete vaccination with either the Pfizer-BioNTech or Moderna vaccine was associated with levels of anti-S IgA positivity greater than 90% with 103 of the 113 (91%) completely vaccinated with the Pfizer-BioNTech vaccine positive for IgA and 214 of the 217 (98%) of those completely vaccinated with the Moderna vaccine (p = 0.0009). One hundred twenty-seven (95%) of the 133 with evidence of prior infection were positive for anti-S IgA as compared to 274 of the 298 (92%) of those without (p = 0.18).

**Figure 1:**
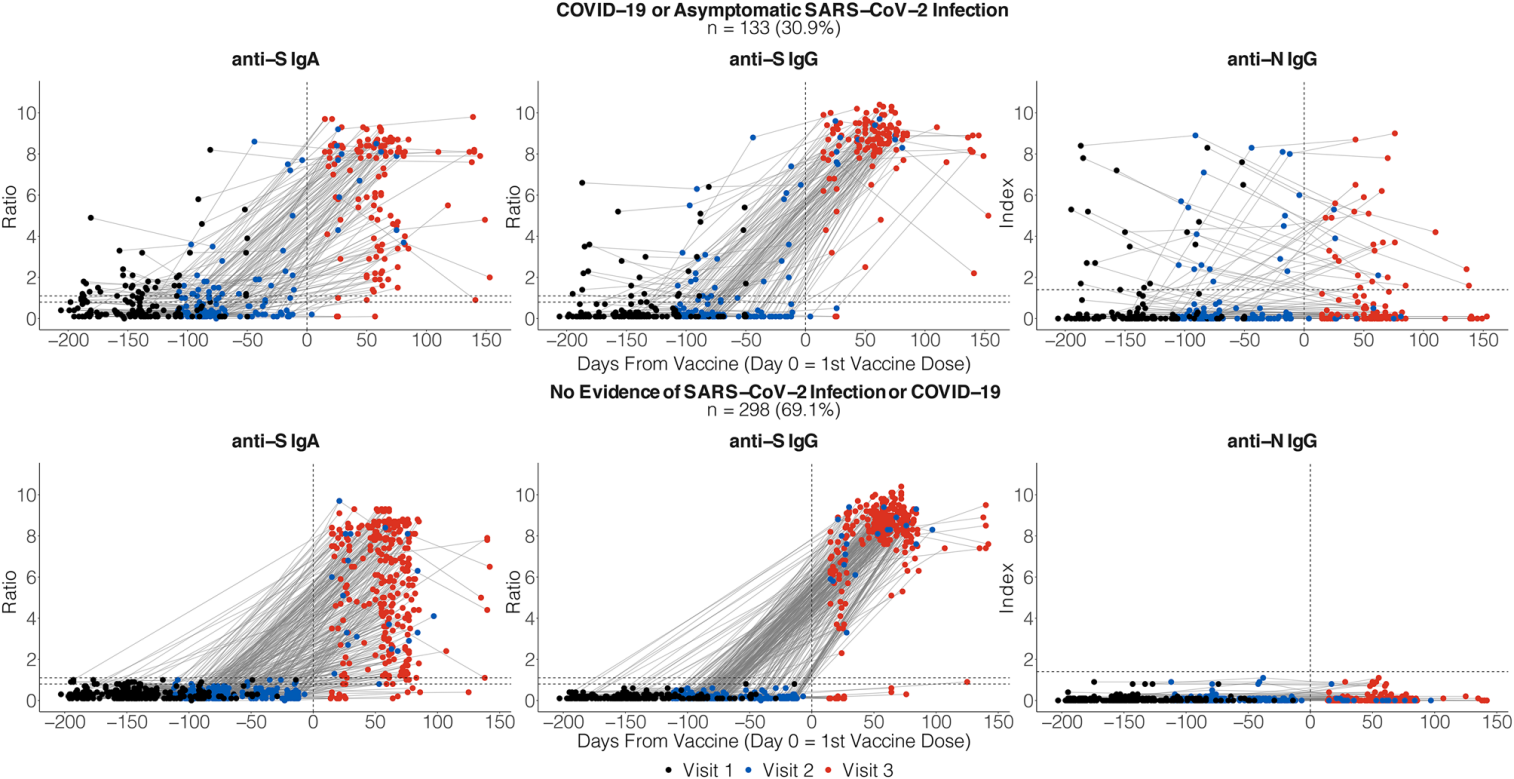
Change in anti-spike IgA ratio, anti-spike IgG ratio and anti-N IgG index relative to time of COVID vaccination comparing those with evidence of prior SARS-CoV-2 infection to those with no evidence of prior infection. Data visualization was performed in R with the ggplot2 package.

Figure 1 demonstrates the trajectory of change in Euroimmun anti-S IgA ratio, Euroimmun anti-S IgG ratio, and Abbott anti-N IgG index relative to the vaccination date comparing the response between the 133 participants with a history of SARS-CoV-2 infection (Definite COVID-19, Probable COVID-19, Possible COVID-19, Definite Asymptomatic Infection) and the 298 participants categorized as No Evidence of SARS-CoV-2 Infection.

## Discussion

Our work demonstrates that the majority of individuals had an elicited IgA response after COVID-19 vaccination in the absence of known prior SARS-CoV-2 infection or COVID-19. While a portion of these individuals may reflect cases of boosting after unrecognized prior infection, the high proportion observed suggests that the vaccines result in a systemic response with both anti-S IgG and anti-S IgA detected in the serum. This is concordant with a smaller prior study demonstrating serum IgA responses to the Pfizer BioNTech and Sinovac inactivated virus vaccines but detectable mucosal responses only in response to the Pfizer BioNTech vaccine^12^.

Plasma IgA is monomeric, typically 90% of which is IgA1 and 10% is IgA2^13^. The distribution of IgA1 and IgA2 in nasal mucosa similar but the percentage of IgA1 found in the saliva is lower at 60%. The specific role of monomeric IgA in the immune response is complex, varying by subtype, with IgA1 playing a potential immunomodulatory role^14^. The presence of IgA in the sera has been associated with detectable levels of IgA in mucosal secretions among those with prior infection^6^. This may reflect a combination of translocation of monomeric IgA from the serum to the mucosa and production of secretory dimeric IgA in the mucosal lymphoid tissue.

Intranasally administered vaccines have been thought to have a potential advantage due to their ability to stimulate a strong mucosal immune response^15^. It is not known the extent to which the observed rise in IgA in the sera following vaccination against SARS-CoV-2 is associated with production and release of secretory IgA. Ketas et al. identified IgA in saliva following immunization against SARS-CoV-2 at a level approximately 1000 times lower than identified in the sera^11^. Chan et al. were able to demonstrate the presence neutralizing antibody in mucosal secretions in the immediate post-vaccination period. However, in a small longitudinal cohort within the same study mucosal-fluid neutralizing activity waned significantly by the time of 3-month follow-up and they were not able to distinguish neutralizing activity specifically attributable to mucosal IgA^12^.

Variability in the ability to elicit an effective IgA response, whether through direct stimulation of secretory IgA production or through translocation from the serum, may offer a potential explanation for the significant variability seen in the ability of COVID-19 vaccines to prevent symptomatic illness and secondary transmission. To further evaluate this association, studies in individuals confirmed to have no prior exposure to SARS-CoV-2 measuring serum and mucosal IgA and distinguishing secretory from potentially translocated monomeric IgA in the mucosal secretions are needed.

## Materials and methods

The study recruited first responders and other personnel from Police, Firefighters, and Emergency Medicine Service (EMS) agencies. Detail regarding recruitment and study participants have been previously published^7^. Participants were surveyed at baseline and follow-up regarding prior exposure to SARS-CoV-2, periods of quarantine from work, symptomatic illness potentially consistent with COVID-19, and confirmed diagnoses of COVID-19.

We categorized individuals based on the serological and clinical evidence of SARS-CoV-2 infection and disease as follows: *Definite COVID-19* includes participants with a positive COVID-19 RT-PCR or antigen test OR a positive IgG antibody combined with a history of exposure to a person with COVID-19 and a history of symptoms compatible with COVID-19; *Probable COVID-19* includes participants with positive IgA or IgG antibody combined with a history of symptoms compatible with COVID-19 OR a history of exposure to a person with COVID-19 AND a history of symptoms compatible with COVID-19; *Possible COVID-19* includes participants with a history of exposure to a person with COVID-19 and a history of symptoms compatible with COVID-19 without positive IgG or IgA antibodies; *Definite Asymptomatic Infection* includes participants with positive IgA or IgG antibody with a history of exposure to a person with COVID-19 with no history of symptoms compatible with COVID-19 OR a positive IgG antibody without history of exposure to a person with COVID-19 and without a history of symptoms compatible with COVID-19; *Possible Asymptomatic Infection* includes participants with positive IgA antibody alone without history of exposure to a person with COVID-19 and without a history of symptoms compatible with COVID-19 and *No Evidence of SARS-CoV-2 Infection or COVID-19* includes participants for whom all antibodies were negative and there was neither a history of exposure to a person with COVID-19 nor a history of symptoms compatible with COVID-19. Participants were categorized based on the last assessment prior to vaccination.

Sera from participants were tested at three timepoints (baseline, 2–3 months, 6 months) at ICON Laboratories using the Abbott Architect Anti-SARS-CoV2 chemiluminescent microparticle immunoassay (MIA) for IgG (anti-nucleocapsid protein, anti-N), Euroimmun Anti-SARS-CoV-2 ELISA for IgG (anti-S1 domain of spike, anti-S), and the Euroimmun Anti-SARS-CoV2 ELISA for IgA (anti-S1 domain of spike). Results for the anti-N IgG were categorized as either positive or negative based on an signal/cut-off index of greater than or equal to 1.4 for positive results. Associations with anti-S IgG and IgA were evaluated based on an optical-density ratio of greater than or equal to 1.1 corresponding to positive tests based on manufacturer specifications.

Vaccination status was defined as complete if the participant had received a full series at least 14 days prior to the collection. Vaccination was categorized as partial if as of 14 days prior to the collection, they had received only 1 of 2 doses. All others categorized as unvaccinated. Individuals who completed a series at least 14 days prior to the 2nd collection were evaluated based on their response at the time of the 2nd collection only.

To assess the impact of prior infection with SARS-CoV-2 on vaccine response, we compared those categorized as Definite COVID-19, Probable COVID-19, Possible COVID-19, or Definite Asymptomatic Infection to those categorized as No Evidence of SARS-CoV-2 Infection or COVID-19. The percent with positive antibodies by type was summarized by vaccine type, vaccine status, and the categorized history of infection or disease.

Data management and statistical analyses were performed using SAS® Version 9.4 (SAS Institute Inc., Cary, NC, USA). Chi-square testing was used to assay differences in antibody positivity based on exposure and illness categories and prior receipt of vaccine. Medians were calculated and non-Wilcoxon rank sum tests were performed to assess for differences in IgG and IgA ratios based on prior history of COVID-19 and vaccine manufacturer. Data visualization was performed in R with the ggplot2 package.

IRB approval was obtained from the Western IRB, protocol # 20201662. Informed consent was obtained from all participants at the time of study recruitment. All study recruitment and laboratory testing were performed in accordance with relevant guidelines and regulations.

## Data Availability

A deidentified dataset including the data used for the analyses may be provided on request to the corresponding author BTM.
